# Design, Synthesis, and Antitumor Activity of Novel Quinazoline Derivatives

**DOI:** 10.3390/molecules22101624

**Published:** 2017-09-28

**Authors:** Liuchang Wang, Pengna Li, Baolin Li, Yawen Wang, Jiangtao Li, Limei Song

**Affiliations:** 1 School of Chemical Engineering, The Key Laboratory for Surface Engineering and Remanufacturing in Shaanxi Province, Xi’an University, Xi’an 710065, China; wlg_112@163.com (L.W.); Lijiangtao-968@126.com (J.L.); slmxawl@163.com (L.S.); 2 School of Chemistry and Chemical Engineering, Shaanxi Normal University, Xi’an 710062, China; baolinli@snnu.edu.cn; 3 College of Chemistry and Chemical Engineering, Taiyuan University of Technology, Taiyuan 030024, China; wangyawen@tyut.edu.cn

**Keywords:** EGFR, tyrosine kinase inhibitor, synthesis, 4-stilbenylamino-4(3*H*)-quinazoline, antitumor agents

## Abstract

In an attempt to explore a new class of epidermal growth factor receptor (EGFR) inhibitors, novel 4-stilbenylamino quinazoline derivatives were synthesized through a Dimorth rearrangement reaction and characterized via IR, ^1^H-NMR, ^13^C-NMR, and HRMS. Methoxyl, methyl, halogen, and trifluoromethyl groups on stilbeneamino were detected. These synthesized compounds were evaluated for antitumor activity in vitro against eight human tumor cell lines with an MTS assay. Most synthesized compounds exhibited more potent activity (IC_50_ = ~2.0 μM) than gefitinib (IC_50_ > 10.0 μM) against the A431, A549, and BGC-823 cell lines. Docking methodology of compound 6c and 6i binding into the ATP site of EGFR was carried out. The results showed that fluorine and trifluoromethyl played an important role in efficient cell activity.

## 1. Introduction

Epidermal growth factor receptor (EGFR) [1,2] belongs to a family of cell surface receptor tyrosine kinases, which is composed of four members: EGFR (HER1 or ErbB-1), HER2 (ErbB-2), HER3 (ErbB-3), and HER4 (ErbB4) [3,4]. The signaling cascade of EGFRs plays a key role in regulating cell proliferation, differentiation, and migration in many tissue types [5,6]. Many of the known tyrosine kinases are integral transmembrane receptors that act to transduce extracellular signals to intracellular responses [7,8,9,10]. Several lines of evidence have implicated that EGFR ligands play a direct role in tumor development and progression [11,12]. The altered protein expression and activity of receptor tyrosine kinase (TK) are implicated in the progression of various types of cancers [13,14]. So, the EGFR tyrosine kinase (EGFR-TK) is an attractive target for the development of agents to direct against tumors which either overexpress EGFR or have a mutated or amplified gene encoding EGFR [15]. “Knocking out” of EGFR-TK activity and the DNA damage induced by the alkylating species are expected to culminate in a sustained antiproliferative activity in EGFR-overexpressing cells.

**Figure 1:**
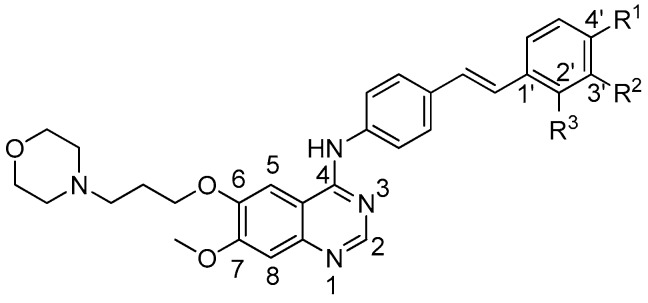
Chemical structures of 4-anilinoquinazoline derivatives **6a**–**6j**.

A series of 4-anilinoquinazoline derivatives have been developed as selective and effective EGFR inhibitors, such as Gefitinib (ZD1839, Iressa) [16,17], erlotinib (OSI-774, Tarceva) [18], and Lapatinib [19,20]. On the basis of the previous investigation, we designed a series of novel 4-substitutedquinazoline derivatives as potential kinase inhibitors. In the quinazoline derivatizations, the thrust of efforts was in the arylamino moiety at the C-4 position of the quinazoline (Figure 1). On the other hand, resveratrol exhibits a variety of biological activities, such as antioxidant, anti-inflammatory, anti-infective, anti-ischemic, cardioprotective, neuroprotective, anti-aging (prolongs lifespan), anti-obesity, and anti-viral activities [21,22]. Especially, resveratrol has been reported as a potential cancer chemopreventive agent for its striking inhibitory effects on cellular events associated with cancer initiation, promotion, and progression [23].

We mainly focused on stilbene analogues, which act as an aryl moiety in the new quinazoline compounds. At the same time, the introduction of stilbene groups to the 4-position of quinazoline would enhance the stability and lipophilicity for the changing of the electronic environment. The activity of the new quinazoline compounds could be improved for a π–π interaction and a CH–π interaction between the benzene ring of stilbene and the aromatic nucleus of target protein residues. Furthermore, the substituent group on stilbene significantly affects the electronic environment of the new quinazoline derivatives [24]. On the other hand, hydrogen bonds play an important role in the interaction of ligands and residues of target proteins. So, as a hydrogen bond acceptor, fluorine was considered to be a high biological activity group [25]. These changes will influence bioavailability, susceptibility to metabolism, and the pharmacological profile of the resulting analogues. We envisioned that chemical modification of the stilbene moiety would further improve the activity of these resulting quinazoline compounds. Therefore, we assumed that a new hybrid incorporating the active parts of each compound might be more potent than any one compound alone. Additionally, this hypothesis could also be checked by a molecular docking methodology.

In this study, we synthesized a series of novel 4-stilbenylamino quinazoline derivatives and investigated the structure-activity relationships (SAR) of these derivatives [26]. The half-maximal inhibitory concentrations (IC_50_) of the synthetic compounds were measured on representative EGFR-positive cell lines: A431, A549, Hela, SMMC-772, BGC823, SK-OV-3, HL-60, and HepG2 by the MTS method. With this assay system, 4-stilbenylamino quinazoline derivatives were proved to be the most promising inhibitor to tumor cells.

It was found that the trifluoromethyl group, and the fluoro and bromo groups on stilbene can improve activity. The Docking results showed that fluorine as a hydrogen bond acceptor, which plays an important role in interactions with residues of target proteins, was considered to be a high biological activity group. On the contrary, the methyl and methoxyl groups contribute less to the increase in activity of the quinazoline derivative.

## 2. Results and Discussion

### 2.1. Chemistry

**Scheme 1:**
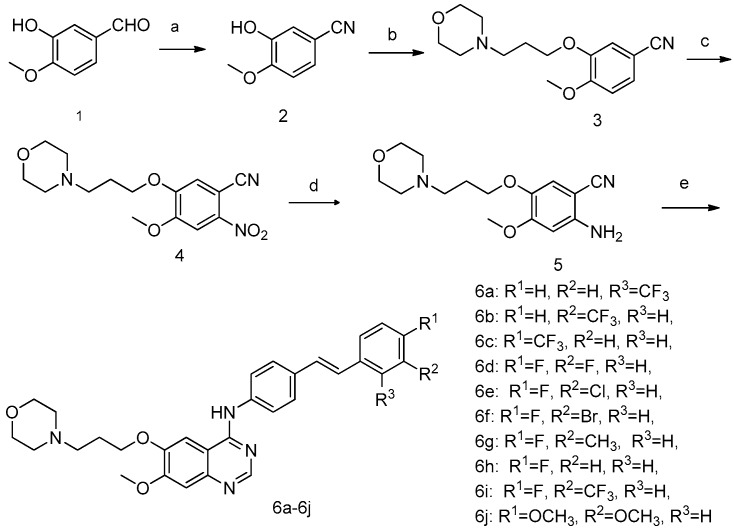
Synthesis of 4-substituted anilino quinazoline derivatives (**6a**–**6j**). Reagents and conditions: (**a**) (HONH_3_)_2_SO_4_, 85 °C, 5 h; (**b**) K_2_CO_3_, DMF, 85 °C, 10 h; (**c**) H_2_SO_4_, HNO_3_, HOAc, room temperature (rt), 50 h; (**d**) Na_2_S_2_O_4_, H_2_O/CH_3_OH, HCl, 50–70 °C, 2 h; (**e**) DMF-DMA, toluene, 105 °C, 3 h, and HOAc, 130 °C, 1.5–3 h.

Scheme 1 outlines the synthetic pathway to prepare compounds **6a**–**6j** through 2-amino-4-methoxy-5-(3-morpholinopropoxy)benzonitrile (**5**), which is a key intermediate. Commercially available isovanillin (3-hydroxy-4-methoxybenzaldehyde) as starting material was reacted with hydroxylamine sulfate in the presence of sodium formate/formic acid to afford 3-hydroxy-4-methoxybenzonitrile (**2**) in 92% yield. The stirring of compound **2** with *N*-(3-chloropropyl)morpholine in DMF with K_2_CO_3_ gave 4-methoxy-3-(3-morpholinopropoxy)benzonitrile (**3**) in 98% yield. The nitration reaction of compound **3** with 70% HNO_3_/70% H_2_SO_4_ (volume ratio = 1:5) provided 4-methoxy-5-(3-morpholinopropoxy)-2-nitrobenzonitrile (**4**) in 84% yield. The key intermediate 2-amino-4-methoxy-5-(3-morpholinopropoxy)benzonitrile (**5**) was obtained by Dimroth rearrangement reaction from compound **4** in the presence of sodium dithionite at 50 °C with a yield of 84%. Compound **5** was treated with dimethylformamide-dimethylacetal (DMF-DMA) and further reacted with substituted 4-aminostilbenes to produce **6a**–**6j** in yields of 41–86%. The substituted (*E*)-4-styrylanilines were obtained from a reduction of substituted (*E*)-4-nitrostilbenes [27] by 80% hydrazine hydrate in present of Fe^+^ in yields of 38–79%.

### 2.2. Biology

#### In Vitro Antitumor Evaluation

The in vitro antitumor abilities of the synthetic compounds were measured on representative EGFR-positive cell lines A431, A549, Hela, SMMC-772, BGC823, SK-OV-3, HL-60, and HepG2 by the MTS method and Geifitinib as a reference drug control [16]. The response parameter (IC_50_) was calculated for each cell line (Table 1). As shown in Table 1, almost all of the synthesized quinazoline derivatives exhibited excellent growth inhibitory activities, with IC_50_ values in the range of 1.23–5.17 μM comparative to Gefitinib IC_50_ (>10.0 μM).

### 2.3. Molecular Modeling

**Figure 2:**
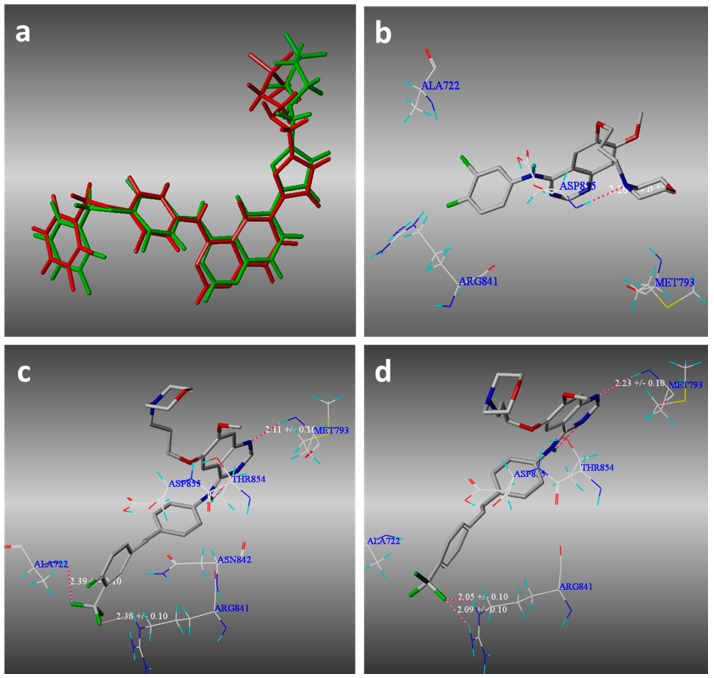
Gradient-optimized pose of Lapatirib (red) versus crystallographically determined structure (green) (**a**). Docking of compounds Gefitinib (**b**), **6i** (**c**), and **6c** (**d**) into the active site of epidermal growth factor receptor (EGFR).

To gain insight into the binding mode of these compounds, molecular docking of the compounds **6c** and **6i** as representative examples of the same series into the binding site of EGFR was carried out. All calculations were performed using SYBYL-X 2.0 software. The crystal structure of EGFR with lapatinib (PDB ID: 1XKK) was obtained from the protein data bank (PDB). Molecular docking studies of the compounds were performed to rationalize the obtained biological results and their mechanism of action (Figure 2). Additionally, molecular docking studies helped us to understand various interactions between the ligands and the enzyme active site in detail.

The results have revealed that the obtained compounds bind to a narrow hydrophobic pocket through the formation of strong hydrogen bond interactions. The complexes were energy-minimized with an AMBER7 FF99 Force-field until a gradient convergence of 0.001 kcal/mol was reached. The docking studies of compounds 6c and 6i showed that a hydrogen bond formed between the N1 of the quinazoline ring and Met-793 (2.23 ± 0.10 Å and 2.11 ± 1.10 Å, respectively). Two hydrogen bonding interactions were observed between CF3−4’ of compound 6c and Arg-841 (2.05 ± 0.10 Å, 2.09 ± 1.10 Å). Different from 6c, CF3-4’ compound 6i formed hydrogen bonding interactions with Ala-722 and Arg-841(2.39 ± 0.10 Å and 2.38 ± 1.10 Å, respectively). The docking studies indicated that the hydrogen bond interactions of the nitrogen atom on the morpholine ring and the NH at the C-4 position of quinazoline with residues in the hinge region of EGFR were uncertain, which hinge on the configuration of compounds and species of substituent groups on the stilbene. These interactions revealed the importance of both fluorine and nitrogen atoms for binding and the subsequent inhibitory capacity. From the above results, we have known that the binding model of compounds 6c and 6i in the ATP binding site were very close to the complex of lapatinib (PDB ID: 1XKK). These molecular docking study results can strongly support our postulation that the synthesized compounds may effectively act on EGFR-TK.

## 3. Experiment Section

### 3.1. General

Unless otherwise stated, all chemicals were purchased form commercial sources and without purification. The ^1^H-NMR (300 MHz) and ^13^C-NMR (75 MHz) spectra, which were recorded using DMSO-*d*_6_ as solvent, were run on a Bruker Avance 300 MHz (Bruker, Bremerhaven, Germany). Chemical shifts (δ) are reported in parts per million, and coupling constants (*J*) are in hertz, using TMS as an internal standard. Data are presented as follows: chemical shift (ppm), multiplicity (s = singlet, d = doublet, t = triplet, q = quartet, m = multiplet). The IR spectra were recorded with a Nicolet Avatar360E.S.P (Bruker, Bremerhaven, Germany). FT-IR spectrometer using KBr pellets. The melting points were determined using an X-5 micro melting point instrument (Beijing Tech., Beijing, China) (the thermometer was not corrected). High resolution mass spectra (HRMS) were performed on a MaXis UHR-TOF (Bruker, Bremerhaven, Germany) with direct injection of the sample.

*3-Hydroxy-4-methoxybenzonitrile* (**2**). A mixture of 3-hydroxy-4-methoxybenzaldehyde (2.0 g, 13.4 mmol), sodium formate (1.8 g, 26.3 mmol), and formic acid (9.6 mL) was heated to 85 °C. Hydroxylamine sulfate (1.3 g, 7.9 mmol) was added to the above mixture in six equal portions at 30 min intervals, and the mixture was stirred for 5 h. The reaction was cooled to room temperature and poured to a solution of sodium chloride (8.0 g) in water (40 mL). The resultant solid was collected by filtration, washed with water, and dried to give an off-white solid **2** (1.8 g, 92%). IR ν_max_ (KBr) cm^−1^: 3311, 3071, 2938, 2231, 1611, 1578, 1511, 1451, 1337, 1284, 1252, 1128, 1020, 952, 860, 810, 610.

*4-Methoxy-3-(3-morpholinopropoxy)benzonitrile* (**3**). A mixture of compound **2** (10 g, 67.1 mmol), K_2_CO_3_ 16.5 g, 4-(3-chloropropyl)morpholine (10.9 g, 66.8 mmol), and DMF 62.5 mL was heated to 85 °C for 10 h. The DMF was removed under vacuum to leave a residue which was partitioned between tert-butyl methyl ether and water. The organic phase was dried by MgSO_4_ and evaporated to give a viscous liquid **3** (18.0 g, 98%). For ^1^H-NMR (300 MHz, DMSO-*d*_6_) δ (ppm): 7.39–7.42 (m, 2H), 7.09–7.12 (m, 1H), 4.06 (s, 2H), 3.86 (s, 3H), 3.59 (s, 4H), 2.11–2.43 (m, 6H), 1.17 (S, 2H).

*4-Methoxy-5-(3-morpholinopropoxy)-2-nitrobenzonitrile* (**4**). Compound **3** (2.6 g, 9.4 mmol) was dissolved in HOAc (6.5 mL) at room temperature. A mixture of H_2_SO_4_ (70%, 6.5 mL) and HNO_3_ (70%, 1.3 mL) was cooled to room temperature, then was added slowly to the above solution in an ice/water bath. The mixture was slowly warmed to room temperature and stirred for 50 h. After an addition of water (40 mL), the mixture was basified to pH 11 with addition of 50% NaOH aqueous solution. CH_2_Cl_2_ was added to the mixture, which dissolved the solid. The aqueous phase was further extracted with CH_2_Cl_2_. The combined organic phase was washed with water, dried by MgSO_4_, and evaporated to give a yellow solid **4** (2.5 g, 84%). For ^1^H-NMR (300 MHz, DMSO-*d*_6_) δ (ppm): 7.86 (s, 1H), 7.70 (s, 1H), 4.29–4.22 (t, 2H), 3.98 (s, 3H), 3.59–3.58 (t, 4H), 2.40–2.38 (m, 6H), 1.94–1.92 (m, 2H).

*2-Amino-4-methoxy-5-(3-morpholinopropoxy)benzonitrile* (**5**). To a suspension compound **4** (2.0 g, 6.2 mmol) in water (30.4 mL), sodium dithionite (3.6 g, 20.7 mmol) was added. The mixture was stirred at 50 °C for 2.5 h. After the mixture was heated to 70 °C, 37% HCl (25 mL) was added slowly in a period of 2 h. Heating was continued for another 1 h. After cooling to room temperature, the mixture was basified to pH 11 with 50% NaOH aqueous solution. The mixture was extracted by CH_2_Cl_2_ for three times. The solution was evaporated, and the residue was purified by silica gel chromatography with eluent (20:1 CH_2_Cl_2_/EtOH) to give a viscous liquid **5** (1.5g, 84%). For ^1^H-NMR (300 MHz, DMSO-*d*_6_) δ (ppm): 6.89 (s, 1H), 6.40 (s, 1H), 5.63 (s, 2H), 3.84–3.88 (t, 2H), 3.73 (s, 3H), 3.56 (m, 4H), 2.35 (m, 6H), 1.77–1.81 (m, 2H). ^13^C-NMR (75 MHz, DMSO-*d*_6_) δ (ppm): 155.4, 148.9, 139.9, 119.1, 116.4, 99.4, 84.2, 68.1, 66.7, 55.8, 55.3, 53.8, 26.5.

(*E)-7-Methoxy-6-(3-morpholinopropoxy)-4-(4-(2-(trifluoromethyl)styryl)phenylamino)quinazoline* (**6a**). A mixture of compound **5** (1.05 g, 3.60 mmol), toluene (9.3 mL), HOAc (0.01 mL), and DMF-DMA (25 mL) was heated to 105 °C and stirred for 3 h. The toluene was completely stripped off under vacuum. To the residue without further purification were added HOAc (9.3 mL) and (*E*)-4-(2-(trifluoromethyl)styryl)aniline (0.95 g, 3.60 mmol). Then, the mixture was heated to 130 °C and stirred for 1.5 h. The HOAc was stripped off under vacuum. To the residue was added water (14.4 mL). The suspension was adjusted to pH 9 with ammonia solution. The precipitate was filtered and wished with ethyl acetate to obtain crude product. The crude product was extracted by dichloromethane, washed with water, and dried by MgSO_4_. The solution was concentrated in vacuo to give off-white solid **6a** (1.53 g, 75% Yield). m.p.: 204.1~206.2 °C. For ^1^H-NMR (300 MHz, DMSO-*d*_6_) δ (ppm): 9.60 (s, 1H), 8.50 (s, 1H), 7.46–8.02 (9H), 7.35 (2H), 7.21 (s, 1H), 4.21 (bs, 2H), 3.95 (s, 3H), 3.59 (bs, 4H), 2.41 (bs, 6H), 2.00 (bs, 2H). For ^13^C-NMR (75 MHz, DMSO-*d*_6_) δ (ppm): 156.6, 156.5, 154.9, 153.2, 148.8, 147.5, 140.4, 136.4, 133.4, 133.2, 131.7, 128.0, 127.6, 127.5, 126.4, 122.7, 122.5, 121.9, 109.5, 107.74, 103.22, 67.64, 66.63, 56.31, 55.44, 53.89, 26.33. IR ν_max_ (KBr) cm^−1^: 3364, 2954, 1620, 1573, 1511, 1428, 1314, 1241, 1155, 1112, 1034, 960, 858, 770. HRMS (ESI-TOF) for C_31_H_31_F_3_N_4_O_3_ [M + H]^+^: Calcd: 564.2348, found: 564.2361.

Compounds (**6b**–**6j**) were prepared from compound **5** and corresponding substituted stilbene following a procedure similar to that described for **6a**.

*(E)-7-Methoxy-6-(3-morpholinopropoxy)-4-(4-(3-(trifluoromethyl)styryl)phenylamino)quinazoline* (**6b**). Yellow solid, Yield 67%. m.p.: 199.1~201.0 °C. For ^1^H-NMR (300 MHz, DMSO-*d*_6_) δ (ppm): 9.57 (s, 1H), 8.50 (s, 1H), 7.87–7.95 (m, 4H), 7.60–7.69 (m, 5H), 7.42 (d, 1H, *J* = 16.4 Hz), 7.34 (d, 1H, *J* = 16.4 Hz), 7.21 (s, 1H), 4.19 (t, 2H), 3.94 (s, 3H), 3.59 (t, 4H), 2.40 (m, 6H), 2.00 (t, 2H). For ^13^C-NMR (75 MHz, DMSO-*d*_6_) δ (ppm): 156.1, 154.4, 152.8, 148.3, 147.0, 139.6, 138.5, 131.5, 130.2, 129.9, 129.7, 126.9, 125.3, 123.5, 122.6, 122.0, 109.0, 107.3, 102.8, 67.2, 66.2, 55.8, 55.0, 53.4, 25.9. IR ν_max_ (KBr) cm^−1^: 3352, 2954, 1597, 1576, 1514, 1425, 1326, 1241, 1124, 1070, 961, 859. HRMS (ESI-TOF) for C_31_H_31_F_3_N_4_O_3_ [M + H]^+^: Calcd: 565.2348, found: 565.2351.

*(E)-7-Methoxy-6-(3-morpholinopropoxy)-4-(4-(4-(trifluoromethyl)styryl)phenylamino)quinazoline* (**6c**). Yellow solid, Yield 60%. m.p.: 219.5~221.2 °C. For ^1^H-NMR (300 MHz, DMSO-*d*_6_) δ (ppm): 9.57 (s, 1H), 8.51 (s, 1H), 7.67–7.92 (9H), 7.27–7.46 (dd, 2H), 7.21 (s, 1H), 4.18–4.22 (t, 2H), 3.95 (s, 3H), 3.57–3.60 (t, 4H), 2.40–2.51 (m, 6H), 1.98–2.02 (m, 2H). For ^13^C-NMR (75 MHz, DMSO-*d*_6_) δ (ppm): 155.6, 154.0, 152.3, 147.8, 146.6, 141.0, 139.3, 130.9, 130.6, 126.9, 126.6, 126.6, 125.1, 125.0, 124.9, 122.1, 121.6, 108.6, 106.8, 102.3, 66.7, 65.7, 55.4, 54.5, 54.4, 53.0, 25.4. IR ν_max_ (KBr) cm^−1^: 3316, 2957, 1596, 1510, 1426, 1321, 1242, 1109, 1064, 958, 858. HRMS (ESI-TOF) for C_31_H_31_F_3_N_4_O_3_ [M + H]^+^: Calcd: 565.2348, found: 565.2355.

*(E)-7-Methoxy-6-(3-morpholinopropoxy)-4-(4-(3,4-difluorostyryl)phenylamino)quinazoline* (**6d**). Yellow solid, Yield 78%. m.p.: 225.8~227.6 °C. For ^1^H-NMR (300 MHz, DMSO-*d*_6_) δ (ppm): 9.55 (s, 1H), 8.50 (s, 1H), 7.12–7.87 (11H), 4.20 (bs, 2H), 3.95 (s, 3H), 3.59 (bs, 4H), 2.40 (bs, 6H), 2.00–2.09 (m, 2H). For ^13^C-NMR (75 MHz, DMSO-*d*_6_) δ (ppm): 156.6, 154.9, 153.2, 148.8, 147.5, 139.9, 135.9, 132.0, 130.1, 127.2, 125.3, 123.8, 122.6, 122.5, 118.2, 118.0, 114.9, 114.7, 109.5, 107.8, 103.3, 67.7, 66.7, 56.3, 55.4, 53.9, 26.4. IR ν_max_ (KBr) cm^−1^: 3319, 2952, 1599, 1517, 1426, 1295, 1240, 1209, 1140, 1111, 961, 857, 819, 612. HRMS (ESI-TOF) for C_30_H_30_F_2_N_4_O_3_ [M + H]^+^: Calcd: 533.2286, found: 533.2292.

*(E)-7-Methoxy-6-(3-morpholinopropoxy)-4-(4-(3-chloro-4-fluorostyryl)phenylamino)quinazoline* (**6e**). Yellow solid, Yield 86%. m.p.: 216.6~219.5 °C. For ^1^H-NMR (300 MHz, DMSO-*d*_6_) δ (ppm): 9.55 (s, 1H), 8.49 (s, 1H), 7.83–7.88 (m, 4H), 7.60–7.63 (3H), 7.38–7.44 (m, 1H), 7.14–7.33 (m, 3H), 4.19 (t, 2H), 3.94 (s, 3H), 3.58 (bs, 4H), 2.39–2.46 (6H), 1.99–2.02 (m, 2H). For ^13^C-NMR (75 MHz, DMSO-*d*_6_) δ (ppm): 158.4, 156.6, 154.9, 153.2, 148.7, 147.5, 139.9, 136.0, 132.0, 130.1, 128.2, 127.2, 125.0, 122.5, 120.3, 117.7, 117.4, 109.5, 107.8, 103.2, 67.6, 66.6, 56.3, 55.4, 53.9, 26.3. IR ν_max_ (KBr) cm^−1^: 3321, 2949, 1621, 1599, 1513, 1426, 1241, 1140, 1111, 1064, 959, 921, 859, 819. HRMS (ESI-TOF) for C_30_H_30_ClFN_4_O_3_ [M + H]^+^: Calcd: 549.1990, found: 549.2006.

*(E)-7-Methoxy-6-(3-morpholinopropoxy)-4-(4-(3-bromo-4-fluorostyryl)phenylamino)quinazoline* (**6f**). Yellow solid, Yield 73%. m.p.: 213.6~215.1 °C. For ^1^H-NMR (300 MHz, DMSO-*d*_6_) δ (ppm): 9.55 (s, 1H), 8.50 (s, 1H), 7.86–7.97 (4H), 7.61–7.63 (3H), 7.14–7.40 (4H), 4.20 (bs, 2H), 3.95 (s, 3H), 3.59 (bs, 4H), 2.40 (6H), 2.00 (bs, 2H). For ^13^C-NMR (75 MHz, DMSO-*d*_6_) δ (ppm): 159.4, 156.6, 154.9, 153.2, 148.8, 147.5, 139.9, 136.3, 132.1, 131.1, 130.1, 127.9, 127.2, 124.9, 122.6, 117.5, 117.2, 109.5, 107.8, 103.3, 67.7, 66.7, 56.3, 55.4, 53.9, 26.4. IR ν_max_ (KBr) cm^−1^: 3321, 2945, 1622, 1600, 1513, 1472, 1426, 1241, 1140, 1111, 958, 859, 818. HRMS (ESI-TOF) for C_30_H_30_BrFN_4_O_3_ [M + H]^+^: Calcd: 593.1485, found: 593.1494.

*(E)-7-Methoxy-6-(3-morpholinopropoxy)-4-(4-(4-fluoro-3-methylstyryl)phenylamino)quinazoline* (**6g**). Yellow solid, Yield 67%. m.p.: 199.9~201.4 °C. For ^1^H-NMR (300 MHz, DMSO-*d*_6_) δ (ppm): 9.54 (s, 1H), 8.49 (s, 1H), 7.85–7.86 (3H), 7.43–7.62 (4H), 7.10–7.20 (4H), 4.18–4.22 (t, 2H), 3.94 (s, 3H), 3.57–3.60 (t, 4H), 2.34–2.47 (m, 6H), 2.27 (s, 3H), 1.98–2.02 (m, 2H). For ^13^C-NMR (75 MHz, DMSO-*d*_6_) δ (ppm): 162.2, 158.9, 156.6, 154.9, 153.3, 148.7, 147.4, 139.5, 134.0,132.5, 129.7, 128.3, 127.0, 126.4, 126.1, 125.0, 124.7, 122.6, 115.8, 115.5, 109.5, 107.7, 103.2, 67.6, 66.6, 56.3, 55.4, 53.9, 26.3, 14.7. IR ν_max_ (KBr) cm^−1^: 3324, 2947, 1599, 1513, 1426, 1239, 1210, 1139, 1112, 960, 855, 819, 606. HRMS (ESI-TOF) for C_31_H_33_FN_4_O_3_ [M + H]^+^: Calcd: 529.2537, found: 529.2549.

*(E)-7-Methoxy-6-(3-morpholinopropoxy)-4-(4-(4-fluorostyryl)phenylamino)quinazoline* (**6h**). Yellow solid, Yield 41%. m.p.: 214.6~216.2 °C. For ^1^H-NMR (300 MHz, DMSO-*d*_6_) δ (ppm): 9.53 (s, 1H), 8.49 (s, 1H), 7.86 (3H), 7.61–7.67 (4H), 7.20–7.24 (5H), 4.18–4.20 (t, 2H), 3.94 (s, 3H), 3.59 (bs, 4H), 2.40 (m, 6H), 1.99 (m, 2H). For ^13^C-NMR (75 MHz, DMSO-*d*_6_) δ (ppm): 163.6, 160.4, 156.6, 154.9, 153.3, 148.8, 147.5, 139.6, 134.4, 132.4, 128.6, 128.5, 127.0, 126.3, 122.6, 116.1, 115.9, 109.5, 107.8, 103.3, 67.7, 66.7, 56.3, 55.5, 53.9, 26.4. IR ν_max_ (KBr) cm^−1^: 3316, 2946, 1596, 1515, 1427, 1385, 1234, 1139, 1063, 959, 858. HRMS (ESI-TOF) for C_30_H_31_FN_4_O_3_ [M + H]^+^: Calcd: 515.2380, found: 515.2394.

*(E)-7-Methoxy-6-(3-morpholinopropoxy)-4-(4-(4-fluoro-3-trifluoromethylstyryl)phenylamino)quinazoline* (**6i**). Yellow solid, Yield 50%. m.p.: 210.2~212.0 °C. For ^1^H-NMR (300 MHz, DMSO-*d*_6_) δ (ppm): 9.55 (s, 1H), 8.50 (s, 1H), 7.86–7.99 (5H), 7.49–7.66 (3H), 7.26–7.41 (2H), 7.20 (s, 1H), 4.18–4.23 (t, 2H), 3.95 (s, 3H), 3.57–3.60 (t, 4H), 2.40–2.51 (m, 6H), 1.98–2.03 (m, 2H). For ^13^C-NMR (75 MHz, DMSO-*d*_6_) δ (ppm): 156.6, 155.0, 153.2, 148.8, 147.5, 140.0, 135.2, 135.2, 132.8, 132.7, 132.0, 130.6, 127.3, 125.1, 125.1, 124.8, 122.5, 122.4, 118.2, 117.9, 109.5, 107.8, 103.3, 67.7, 66.7, 56.3, 55.4, 53.9, 26.4. IR ν_max_ (KBr) cm^−1^: 3357, 2939, 1619, 1597, 1515, 1424, 1390, 1326, 1241, 1140, 1053, 959, 921, 848. HRMS (ESI-TOF) for C_31_H_30_F_4_N_4_O_3_ [M + H]^+^: Calcd: 583.2254, found: 583.2268.

*(E)-7-Methoxy-6-(3-morpholinopropoxy)-4-(4-(3,4-dimethoxystyryl)phenylamino)quinazoline* (**6j**). Yellow solid, Yield 50%. m.p.: 176.1~179.3 °C. For ^1^H-NMR (300 MHz, DMSO-*d*_6_) δ (ppm): 9.52 (s, 1H), 8.49 (s, 1H), 7.86 (s, 1H), 7.83 (d, 2H, *J* = 8.4 Hz), 7.61 (d, 2H, *J* = 8.4 Hz), 7.08–7.25 (5H), 6.97 (d, 1H, *J* = 8.3 Hz), 4.18–4.20 (t, 2H), 3.94 (s, 3H), 3.84 (s, 3H), 3.78 (s, 3H), 3.59 (bs, 4H,), 2.40–2.46 (6H), 1.98–2.02 (m, 2H). For ^13^C-NMR (75 MHz, DMSO-*d*_6_) δ (ppm): 156.1, 154.4, 152.8, 149.0, 148.6, 148.2, 146.9, 138.7, 132.4, 130.3, 127.0, 126.2, 126.0, 122.2, 119.7, 111.9, 109.2, 109.0, 107.3, 102.8, 67.2, 66.2, 55.8, 55.5, 54.9, 53.4, 25.9. IR ν_max_ (KBr) cm^−1^: 3435, 2625, 1589, 1514, 1428, 1243, 1143, 1017, 849. HRMS (ESI-TOF) for C_32_H_36_N_4_O_5_ [M + H]^+^: Calcd: 557.2686, found: 557.2700.

### 3.2. Biological Evaluation of Anti-Tumour of the Synthesized Compounds

The A431 (human vaginal epidermoid cancer cell line), A549 (human non-small cell lung cancer), Hela (human cervical cancer cell line), SMMC-7721 and HepG2 (human liver cancer cell line), BGC-823 (human poorly differentiated gastric cancer cell line), SK-OV-3 (human ovarian cancer cell), HT-29 (human colon cancer cell), and HL-60 (human leukemia cell line) were obtained from the Shanghai Chinese Academy of Sciences Cell Library.

The inhibitory activities of all compounds were evaluated using a commercial viability assay (CellTiter 96^®^ Aqueous One Solution Assay, Corning Corporation) [28]. Typically, the suspension (100 μL/well) with evaluated cells (2.5~4 × 10^4^ cell/mL) and pancreatic protease digestive juice (0.25%) was seeded into 96-well plates. After a 24 h incubation period in CO_2_, media were replaced with DMSO solution (20 μL/well) of the tested compounds (C = 10 μmol/L). Cells were cultured for 48 h with 80 μL of 10% foetal bovine serum (FBS) added. At the end of the incubation time, MTS reagent 100 μL/well (20 μL MTS in 100 μL PBS) was added to each well. After a further incubation of 4 h, the optical density (OD) of each well was read at 490 nm using an Enzyme-linked immunosorbent detector. The assays were performed in triplicate. Results were expressed as percent change from control non-treated cultures. The inhibitory concentrations 50 (IC_50_) were calculated as the concentrations of the compounds required to cause a 50% reduction of absorbance values.

### 3.3. Molecular Modeling Methods

The calculations were performed using SYBYL-X2.0 software installed on 3.30 GHz Core 4 Duo, 8G memory, with a Windows Server 2008 R2 operating system. The crystal structure of lapatinib was obtained from the Protein Data Bank (PDB codes: 1XKK) in order to prepare the protein for docking studies. The preparation of protein structure used the Protonate 3D function, and the molecules were energy-minimized according to the implemented AMBER7 FF99 force-field. The docking procedure followed was the standard protocol implemented in SYBYL-X2.0, and the geometry of the resulting complexes was studied using the pose Viewer utility.

## 4. Conclusions

Herein, we designed and synthesized a series of novel 4-aminoquinazolines derivatives containing a stilbene moiety. Addtionally, the biological activity of these compounds was investigated in several cell lines, including human vaginal epidermoid cancer cell line (A431), human non-small cell lung cancer (A549), and human liver cancer cell line (HepG2). In all of the compounds, fluorine atoms on the stilbene moiety enhanced interaction with residue, which helped with fitting into the lipophilic back pocket, as expected by our designs, leading to an improvement in antiproliferative activity. Most derivatives showed better biological activity and potency (IC_50_ < 2.0 μM) with respect to gefitinib assessed in a series of cell lines. The good growth inhibition of these compounds may owe to the fact that they can more closely bind in the ATP binding site of EGFR, resulting from docking model compounds **6c** and **6i** with 1XKK.

## Figures and Tables

**Table 1:** IC_50_ values of compounds **6a**–**6j** ^a^ and Gefitinib against eight tumor cell lines (μg/mL).

Compounds	A431	A549	Hela	HL-60	SMMC-7721	BGC823	SK-OV-3	HepG2
**6a**	1.55 ± 0.10	1.40 ± 0.13	3.30 ± 0.06	2.281 ± 0.29	3.91 ± 0.66	-	3.34 ± 0.37	5.175 ± 0.95
**6b**	1.81 ± 1.15	2.61 ± 0.89	2.89 ± 0.38	not fit	2.46 ± 0.19	-	2.54 ± 0.14	4.579 ± 0.83
**6c**	1.23 ± 0.36	1.44 ± 0.52	1.68 ± 0.32	2.675 ± 0.20	1.73 ± 0.04	-	1.68 ± 0.07	1.829 ± 0.28
**6d**	2.35 ± 0.85	2.17 ± 0.29	2.64 ± 0.93	2.62 ± 0.08	3.56 ± 1.01	1.32 ± 0.29	2.65 ± 0.87	3.04 ± 0.17
**6e**	2.50 ± 0.25	2.19 ± 0.15	2.63 ± 0.67	2.99 ± 0.55	3.11 ± 0.60	1.82 ± 0.52	3.11 ± 0.58	3.15 ± 0.29
**6f**	1.74 ± 0.20	2.37 ± 0.29	1.96 ± 0.12	2.63 ± 0.06	2.90 ± 0.73	1.59 ± 0.74	2.71 ± 0.52	3.16 ± 0.42
**6g**	1.96 ± 0.98	3.18 ± 1.03	3.05 ± 0.10	2.59 ± 0.02	4.21 ± 0.63	1.38 ± 0.16	3.70 ± 0.79	3.94 ± 1.56
**6h**	2.02 ± 0.80	2.22 ± 0.82	1.74 ± 0.44	2.49 ± 0.19	2.34 ± 0.67	1.27 ± 0.16	2.77 ± 0.68	2.79 ± 0.19
**6i**	1.27 ± 0.95	1.67 ± 0.38	3.77 ± 0.63	3.11 ± 0.01	5.37 ± 0.02	1.66 ± 0.38	5.26 ± 1.30	3.98 ± 0.88
**6j**	2.71 ± 0.17	3.82 ± 0.80	-	1.73 ± 0.47	3.36 ± 0.93	2.13 ± 0.85	2.18 ± 0.97	2.68 ± 0.59
Gefitinib ^b^	12.93 ± 4.54	13.75 ± 5.73	17.92 ± 1.50	17.72 ± 1.76	23.27 ± 0.66	>10	12.31 ± 0.33	>10

^a^ All biological data are mean values for three independent experiments performed in duplicate; ^b^ Geifitinib purchased from commercial sources was used as positive control.
